# Supplementary material for: COZOID: contact zone identifier for visual analysis of protein-protein interactions
Source: BMC Bioinformatics. 2018 Apr 6;19:125. doi: 10.1186/s12859-018-2113-6 (PMC5889581; doi:10.1186/s12859-018-2113-6)
Supplement: Supplementary file 2 — Software build. Executable binary file of the software tool. (ZIP 118,784 kb) [file 12859_2018_2113_MOESM2_ESM.zip › cozoid/platform/docs/testng-6.5.1-javadoc.zip!/org/testng/IAnnotationTransformer2.html]

IAnnotationTransformer2 (TestNG)


---


|  |  |  |
| --- | --- | --- |
| **Overview** \| **Package** \| **Class** \| **Tree** \| **Deprecated** \| **Index** \| **Help** | |  |
| **PREV CLASS**   **NEXT CLASS** | **FRAMES**    **NO FRAMES**     **All Classes** |  |
| SUMMARY: NESTED \| FIELD \| CONSTR \| METHOD | DETAIL: FIELD \| CONSTR \| METHOD |  |


---


## org.testng Interface IAnnotationTransformer2

**All Superinterfaces:**: IAnnotationTransformer, ITestNGListener

---

``` public interface IAnnotationTransformer2 extends IAnnotationTransformer ```

Use this interface instead of IAnnotationTransformer if you want to modify any TestNG
annotation besides @Test.

---

| **Method Summary** | |
| --- | --- |
| `void` | `transform(IConfigurationAnnotation annotation, java.lang.Class testClass, java.lang.reflect.Constructor testConstructor, java.lang.reflect.Method testMethod)`             Transform an IConfiguration annotation. |
| `void` | `transform(IDataProviderAnnotation annotation, java.lang.reflect.Method method)`             Transform an IDataProvider annotation. |
| `void` | `transform(IFactoryAnnotation annotation, java.lang.reflect.Method method)`             Transform an IFactory annotation. |

**Methods inherited from interface org.testng.IAnnotationTransformer**

`transform`

**Method Detail**

### transform

```
void transform(IConfigurationAnnotation annotation,
               java.lang.Class testClass,
               java.lang.reflect.Constructor testConstructor,
               java.lang.reflect.Method testMethod)
```

:   Transform an IConfiguration annotation.
    Note that only one of the three parameters testClass,
    testConstructor and testMethod will be non-null.

    :   **Parameters:**: `annotation` - The annotation that was read from your test class.: `testClass` - If the annotation was found on a class, this parameter represents this class (null otherwise).: `testConstructor` - If the annotation was found on a constructor, this parameter represents this constructor (null otherwise).: `testMethod` - If the annotation was found on a method, this parameter represents this method (null otherwise).

---


### transform

```
void transform(IDataProviderAnnotation annotation,
               java.lang.reflect.Method method)
```

:   Transform an IDataProvider annotation.

    :   **Parameters:**: `method` - The method annotated with the IDataProvider annotation.

---


### transform

```
void transform(IFactoryAnnotation annotation,
               java.lang.reflect.Method method)
```

:   Transform an IFactory annotation.

    :   **Parameters:**: `method` - The method annotated with the IFactory annotation.


---


|  |  |  |
| --- | --- | --- |
| **Overview** \| **Package** \| **Class** \| **Tree** \| **Deprecated** \| **Index** \| **Help** | |  |
| **PREV CLASS**   **NEXT CLASS** | **FRAMES**    **NO FRAMES**     **All Classes** |  |
| SUMMARY: NESTED \| FIELD \| CONSTR \| METHOD | DETAIL: FIELD \| CONSTR \| METHOD |  |


---
